# Asymmetric Effect of Business Cycles on Population Health: Evidence From the ASEAN Countries

**DOI:** 10.3389/fpubh.2020.00032

**Published:** 2020-02-21

**Authors:** Yi-Hui Liu, Wen-Hsin Huang

**Affiliations:** ^1^Department of Senior Citizen Service Management, National Taichung University of Science and Technology, Taichung, Taiwan; ^2^Department of Accounting Information, National Taichung University of Science and Technology, Taichung, Taiwan

**Keywords:** asymmetry, business cycles, ASEAN, hidden cointegration, panel vector error correction model

## Abstract

This study investigates the asymmetric effects of business cycles (measured by real GDP per capita) on population health (measured by life expectancy at birth) from the ASEAN countries, namely, Brunei, Cambodia, Indonesia, Laos, Malaysia, Myanmar, Philippines, Singapore, Thailand, and Vietnam. The panel vector error correction model, together with various panel unit root tests and cointegration tests, suggested a hidden cointegrated relationship between life expectancy at birth and the positive and negative components of real GDP per capita, and the asymmetric effects of business cycles on population health were identified in both in the short run and in the long run. Policymakers should focus on the harmful effects of business cycles on population health, and government interventions should be more forceful in times of economic expansion than during periods of economic recession.

## Introduction

Previous studies provide mixed evidence on the effect of business cycles on population health. It is believed that countries with more advanced levels of economic development tend to have higher education levels, better health systems, and individuals are able to afford better health-relevant commodities, all of which are likely to improve health outcomes. Pritchett and Summers (1), in their influential study, proposed that “wealthier is healthier.” The seminal studies conducted by Brenner (2–4) indicated that economic recessions lead to negative effects on health and concluded that mortality is counter-cyclical with respect to business cycles. However, some researchers have found that an economic downturn does not necessarily give rise to poor health. Ruhm (5–7), Neumayer (8), Tapia Granados (9), Gerdtham and Ruhm (10), Lin (11), Gonzalez and Quast (12), Ariizumi and Schirle (13), and Tapia Granados and Ionides (14) all documented that mortality decreased during economic recessions. The concern of the existence of feedbacks from health to economic development has been confirmed by empirical evidence. There is a growing consensus that improving health can directly or indirectly accelerate economic growth in various ways (15–20). Furthermore, Bloom and Canning (18), Tapia Granados and Ionides (21), and Chen (22) found bio-directional causality between business cycles and population health.

While numerous studies have investigated the relationship between business cycles and population health, the mixed empirical evidence deserves further analysis. In this study, we select the countries of the Association of Southeast Asian Nations (ASEAN), namely Brunei, Cambodia, Indonesia, Laos, Malaysia, Myanmar, Philippines, Thailand, and Vietnam, as our research sample. The ASEAN countries have been referred as to emerging economies in recent years. This region also presents a compelling proposition for businesses seeking growth opportunities. ASEAN countries command significant economic weight. The GDP of the region has more than quadrupled since 1999, from US$577 billion in 1999 to US$2.5 trillion in 2016, making it the sixth-largest economy in the world (23). The ASEAN region has experienced several phases of economic development: rapid economic growth, the Asian financial crisis, and economic recovery, and it is beneficial for us to assess the effects of the different economic expansions and recessions. An investigation into ASEAN countries will provide a more complete picture of the relationship between population health and business cycles. We use GDP per capita and life expectancy at birth to proxy business cycles and population health in the ASEAN countries between 1950 and 2016. The data was obtained from the Maddison Project Database 2018 and the Gapminder database.

In this study, we aim to examine the ambiguous relationship between business cycles and population health and to identify the possible existence of asymmetric effects of business cycles on population health. That is, the effect of economic expansions on population health may differ from the effect of business recessions. After real GDP per capita and life expectancy at birth were decomposed by the positive and negative components based on Hatemi (24) and Hatemi and El-Khatib (25), we employ cointergration tests and estimate a panel vector error correction model to observe both the short-run and long-run asymmetric effects between business cycles and population health. Further, based on pairwise Granger causality tests, we utilize impulse-response analyses to observe the short-run asymmetric effects of economic expansions and recessions on population health. The asymmetric effects of business cycles on various economic variables have been studied in the field of macroeconomics, but there are few studies which focus on this in the context of population health.

In order to fill this gap in the literature, the purpose of this study is to investigate the asymmetric effect of business cycles on population health across ASEAN countries from the period of 1950–2016. This study produced two main findings. First, the panel cointegration tests suggest a hidden cointegrated relationship between life expectancy at birth and the positive and negative components of real GDP per capita. The positive component of real GDP per capita has a significantly positive impact on life expectancy at birth, but the negative component of real GDP per capita generates a non-significant effect on life expectancy at birth in the long run. Second, the impulse-response analyses show that an increase in real GDP per capita (i.e., an increase in the positive component of real GDP per capita) will first increase, and then decrease, population health, and a decrease in real GDP per capita (i.e., a decrease in the negative component of real GDP per capita) will decrease population health in the short run. The negative effect of a decrease in real GDP per capita exceeded the positive effect of an increase in real GDP per capita in the short run.

This study contributes the literature of business cycles on population health in two aspects: First, we applied the hidden cointegration methodology proposed by Hatemi (26) to examine the asymmetric effect of business cycles on population health across ASEAN countries for the first time. Second, the asymmetric effects of business cycles on population health were identified to reconcile the ambiguous results from previous studies on the effect of business cycles on population health. The major limitation is the bivariate type of analyses due to the unavailability of other socio-economic variables with complete long-time series data across the ASEAN countries. Nevertheless, the ASEAN share the similar socio-economic development, the impact of limitation on our results should be minor.

The study produced two main findings. First, the panel cointegration tests suggest a hidden cointegrated relationship between life expectancy at birth and the positive and negative components of real GDP per capita. The positive component of real GDP per capita has a significantly positive impact on life expectancy at birth, but the negative component of real GDP per capita generates a non-significant effect on life expectancy at birth in the long run. Second, the impulse-response analyses show that an increase in real GDP per capita (i.e., an increase in the positive component of real GDP per capita) will first increase, and then decrease, population health, and a decrease in real GDP per capita (i.e., a decrease in the negative component of real GDP per capita) will decrease population health in the short run. The negative effect of a decrease in real GDP per capita exceeded the positive effect of an increase in real GDP per capita in the short run.

This study is organized as follows. Section Literature Review provides a review of the existing literature. Section Models and Data describes the empirical models and data used in the study. Section Results presents the empirical results, and Section Discussion discusses the results in more detail. Section Conclusion provides concluding remarks.

## Literature Review

The relationship between business cycles and population health has long been of great concern. Previous research conducted over several decades from a variety of countries and time periods has been rather mixed and inconclusive. Empirical findings have found that the correlation could be positive, negative, or unrelated. As to the direction of causality, some researchers suggest that economic conditions affect population health, others contend that population health affects economic development, and still others point out the existence of bi-directional causality.

The seminal studies conducted by Brenner (2–4) indicated that economic recessions lead to negative effects on health by increasing aggregate mortality rates and deaths from various causes and concluded that mortality is counter-cyclical with respect to business cycles. Following this line of time-series research, some researchers confirmed Brenner's opinions (27–29). However, other researchers criticized Brenner's empirical methodology for model misspecification, lag structure, omitted variable bias, and other statistical problems (30, 31). Subsequent studies trying to address the shortcomings of Brenner's analysis still failed to find analogous results in a replication of his works on other countries or time periods (31–35).

To deal with problems of time series analysis, recent studies on population health and business cycles have turned toward panel data. For example, Ruhm (5–7) used fixed-effect models with state-level observations in the United States and found that mortality behaves pro-cyclically as it moves with the business cycle. The pro-cyclicality of mortality has been documented using data from individual countries such as Germany (8), Spain (9), Mexico (12), and Canada (13), as well as country groupings from Europe (14), the Asia-Pacific region (11), and OECD member countries (10).

However, some studies have observed that the cyclicality of population health seems to be changing with the inclusion of more recent data. Tapia Granados and Ionides (21) showed that the positive relationship between economic growth and population health in Sweden during the nineteenth century became gradually weaker with the passing of time and was completely reversed by the end of the twentieth century, when economic growth negatively affected population health progress. Using data for England and Wales during 1840–2000, Tapia Granados (36) found a negative relationship between the GDP growth rate and the annual increase in life expectancy at birth. This negative effect was much stronger for 1900–1950 than for 1950–2000 and was very weak in the nineteenth century. Ruhm (37) indicated that total mortality in the US shifted from being strongly pro-cyclical to being weakly related to business cycles over the 1976–2010 period. In contrast to aforementioned studies, Gerdtham and Johannesson (38) found that mortality for males (but not females) increased significantly during periods of recession in Sweden. Svensson (39) found that there was no significant relationship between mortality and macroeconomic conditions in Swedish regions. Instead, he observed that the business cycle effect was counter-cyclical related to mortality rates for individuals of prime-working age (between 20 and 49).

The mixed effects of business cycles on population health could stem from the fact that macroeconomic conditions affect health through different channels that could act in opposite directions. From a health behavior-pathway perspective, Ferreira and Schady (40) and Gonzalez and Quast (12) proposed contradictory income and substitution effects on health behaviors. The income effect states changes in the ability of individuals to afford healthcare goods or services and the substitution effect reflects changes in the opportunity cost of health-related activities relative to work. That is to say, economic recessions lead to changes in health behaviors due to the lower income effect and, simultaneously, the increase in the substitution effect. Thus, the total effect of economic circumstances on health outcomes will depend on whether the income or substitution effects dominate.

It is difficult to interpret the relationship between population health and economic development as simply the causal effect of economic development on health. Intuitively, a healthier population is more productive so it is plausible that population health enhances economic development. There is a growing consensus that improving health can directly or indirectly accelerate economic growth in various ways (15–20). Weil (41) concluded that health is an important determinant of economic development and observed that health, when compared with physical or human capital, could help explain a larger magnitude of GDP variance across countries. While many studies have observed the positive effects of health on economic growth, Acemoglu and Johnson (42) found no evidence that the increase in life expectancy lead to a significant increase in income per capita. Their interpretation of the insignificant effect was that other production factors, such as capital and land, did not adjust with population growth.

From a theoretical perspective, there is good reason to believe that causality between business cycles and population health could run in both directions. Healthier individuals are more productive and contribute to creating richer economies. Likewise, higher incomes for individuals or countries improve health in a variety of ways, ranging from better nutrition to the construction of public health infrastructure (43). Empirically, some studies provide evidence that both forms of causality are operative. Bloom and Canning (18) proposed that health improvements lead economic growth, and as population health fosters economic growth, a positive feedback effect could lead to a beneficial situation where population health and macroeconomic conditions are mutually reinforcing.

To capture the two-way interplay between health and economic growth, Barro (44) constructed a theoretical framework with an extension of the neoclassical growth model that considered not only the causality from health status to economic growth, but also the reverse channel from economic growth to health status. The bi-directional causality was also observed in Chen's (22) empirical research. Chen (22) conducted a continuous wavelet analysis to investigate the relationship between life expectancy and GDP per capita in the US. He identified the one-way causal effects from economic growth to population health and from health to economic growth, and therefore identified a bi-directional relationship between economic growth and population health. He concluded that none of the above relationships prevailed over the entire research period. Tapia Granados and Ionides (21) showed the changing relationship between economic growth and change in life expectancy in Sweden. However, the reverse effects of health progress on economic growth was weak and unobservable in the late twentieth century.

The asymmetric effects of business cycles on various economic variables have long been investigated in macroeconomics studies [e.g., (45–51)]. Mocan and Bali (50) found the asymmetric response of crime to changes in the unemployment rate. Katrakilidis and Trachanas (47) identified the asymmetric effects of inflation shocks on stock market prices in Greece. Chang and Chen (45) and Lin and Chen (48) proposed that the relationship between economic development (expansion or recession) and suicide rates is asymmetric for different age groups. To the authors' best knowledge, this study may be the first study to analyze the short-run and long-run asymmetric relationship between business cycles and population health across the ASEAN countries, therefore filling an existing gap in the literature.

## Models and Data

### Empirical Model

The main purpose of this study is to investigate the effect of business cycles on population health in the ASEAN countries. Previous studies such as Liu et al. (49), Chen et al. (52), Lin and Chen (48), Chang and Chen (45), and Chen (22) have investigated the asymmetric relationship between business cycles and population health for certain advanced economies. However, in this study, we follow Hatemi's (26) methodology of using a panel cointegration model to attempt to identify any asymmetric effects of business cycles on population health in emerging economies.

Based on conventional model specification for cointegration analyses proposed by Johansen and Juselius (53), the cointegrated relationship between population health (measured by life expectancy at birth) and economic development (measured by real GDP per capita) in terms of panel vector error correction model (PVECM, hereafter) could be specified as follows:

(1) $$\begin{array}{l} \Delta Y_{it}=C+\alpha \beta^{\prime }\;Y_{it-1}+\sum_{j=1}^{p}\Pi_{j}\Delta Y_{it-j}+\xi_{it} \end{array}$$

where the subscript i (= 1, 2, 3, …, *N*) and *t* (= 1, 2, 3, …, *T*) represent individual country i and the specific year t, respectively. **Δ** is the difference operator and *p* is the lag period identified by the Bayesian information criterion (BIC). **C** denotes a vector of constant term, and **ξ**_**t**_ is the residual vector. ${\text{Y}}_{\text{i}\text{t}}=\left[\ln\;{\left(LE\right)}_{it},\ln\;{\left(GDP\right)}_{it}^{+},\ln\;{\left(GDP\right)}_{it}^{-}\right]$ or ${\text{Y}}_{\text{i}\text{t}}=\left[\ln\;{\left(GDP\right)}_{it},\ln\;{\left(LE\right)}_{it}^{+},\ln\;{\left(LE\right)}_{it}^{-}\right]$. Note that $\ln\;{\left(GDP\right)}_{it}^{+}$ and $\ln\;{\left(GDP\right)}_{it}^{-}$ ($\ln\;{\left(LE\right)}_{it}^{+}$ and $\ln\;{\left(LE\right)}_{it}^{-}$) are positive and negative cumulative sum of economic (health) shocks, respectively. These two cumulative sum of economic (health) shocks were obtained by the decomposition procedure introduced by Hatemi (24) and Hatemi and El-Khatib (25). We describe this technical procedure in the Appendix for the brief presentation of our empirical model. In addition, **Π**_*j*_ and **αβ′** are vector coefficients, and **Π**_*j*_ represents the short-run effect. **α** is adjustment vector indicating the speed of short-run adjustment to long-run equilibrium, and **β** is a cointegrating vector with dimensions *k*x *r* (variables × number of cointegrated relationships). It is important to note that Equation (5) below is the error correction vector (**ec**_**it**_),

(2) $$\begin{array}{l} {\text{β}}^{\prime }\;Y_{it-1}\;=\;ec_{it} \end{array}$$

where the definition of **Y**_**it**−1_ is the same as that in Equation (1), **β′** = [1, −β^0^, −β^*T*^, −β^+^, −β^−^]. Hence, the long-run relationship among the variables of interest can be written as follows:

(3) $$\begin{array}{l} \{\begin{array}{l} \ln\;{\left(LE\right)}_{it}=\beta^{0}+\beta^{T}t+\beta^{+}\ln{\left(GDP\right)}_{it}^{+}+\beta^{-}\ln{\left(GDP\right)}_{it}^{-}+ec_{it}\\ \ln\;{\left(GDP\right)}_{it}=\beta^{0}+\beta^{T}t+\beta^{+}\ln{\left(LE\right)}_{it}^{+}+\beta^{-}\ln{\left(LE\right)}_{it}^{-}+ec_{it} \end{array} \end{array}$$

It is important to address that Equations (1,2,3) are capable of justifying the asymmetric effect of business cycles on population health because we incorporate positive and negative cumulative sum of economic (health) shocks into our empirical model.

Since the negative cumulative sum of shocks were negative, the increase of negative cumulative sum of shocks means a decrease in the negative components of the variables. Therefore, the negative (positive) sign of the β^−^coefficient should be interpreted in such a way that a 1% decrease in the negative component of the variables due to negative shocks would lead to an increase (decrease) of β^−^% in the dependent variables. We proceed with our analyses using the following empirical procedures:

Step 1: Breitung *t*-test (54) was used for unit root null hypothesis testing so that the order of integration of the variables could be identified.
Step 2: If all variables used in our analyses were justified as I(1) time series, then the Kao panel cointegration test would be used to test for null hypothesis of non-existence of cointegration (55). If the null hypothesis of non-existence of cointegration were rejected, the Johansen Fisher panel cointegration test would be used to identify the number of cointegration relationships among variables of interest (56). This is a sequential testing procedure. The Kao panel cointegration test could only testing whether or not there exists co-integrating relation among all variables used in this study. It is possible to find more than one co-integrating relation among variables of interest, and therefore, the Johansen Fisher panel cointegration test would be employed to identify the number of cointegration relationships among variables of interest.
Step 3: Based on the number of cointegration relationships among variables of interest identified by the Johansen Fisher panel cointegration test, we could estimate a PVECM that would reveal the long-run relationship among variables of interest and the short-run adjustment to long-run equilibrium.
Step 4: Pairwise Granger causality tests were used to identify one-way or two-way causal relationships among variables of interest so the directions of the impulse-response analyses could be justified.
Step 5: Based on the results of pairwise Granger causality tests, we explore the short-run effects of business cycles on population health.

### Hypotheses

There are three hypotheses relating to our empirical procedures:

Hypothesis 1 (Pro-cyclicality of population health to business cycles hypothesis):
National income positively Granger causes population health. Namely, the economic growth leads to an increase of population health. It is a sequential procedure to validate this hypothesis: we first applied the F test to investigate the null hypotheses of positive income shocks [H_0_: Δln(GDP)^+^ ≠ > Δln(LE)] and negative income shocks [Δln(GDP)^−^ ≠ > Δln(LE)] leads to population health, and we observe the impulse-response plots to see whether or not the population health responds to the unexpected positive (negative) income shocks positively (negatively).
Hypothesis 2 (Counter-cyclicality of population health to business cycles hypothesis):
National income negatively Granger causes population health. Namely, the economic growth leads to a decrease of population health. Similar to the test procedure used in Hypothesis 2 (Pro-cyclicality of population health to business cycles hypothesis), we first applied the F test to investigate the null hypotheses of positive income shocks [H_0_:Δln(GDP)^+^ ≠ > Δln(LE)] and negative income shocks [Δln(GDP) ^−^ ≠ > Δln(LE)] leads to population health, and we observe the impulse-response plots to see whether or not the population health responds to the unexpected positive (negative) income shocks negatively (positively).
Hypothesis 3 (Asymmetric effect of health cycles hypothesis):
The business cycles have asymmetric effects on population health. The Wald tests were used to test the null hypotheses of the long-run (i.e., H_0_:β^+^ = β^−^) and short-run (i.e., H_0_: $\pi_{\Delta \ln\;\left(GDP\right),t-1}^{+}+\pi_{\Delta \ln\;\left(GDP\right),t-2}^{+}=\pi_{\Delta \ln\;\left(GDP\right),t-1}^{-}+\pi_{\Delta \ln\;\left(GDP\right),t-2}^{-}$) symmetric effects of business cycles on population health.

Hypothesis 1 (Pro-cyclicality of population health to business cycles hypothesis) is closely related to the economic growth theory of human capital. It hypothesizes economic growth increases accumulation of health capital that benefits population health (41). Nevertheless, Hypothesis 2 (Counter-cyclicality of population health to business cycles hypothesis) is drawn from the pathway of individual health behavior that reflects the contradictory income and substitution effects on individuals (22). Namely, population health is counter-cyclical with respect to business cycles, when the substitution effect (meaning the opportunity cost of health-promoting activity relative to work) were higher (lower) than income effect (referring to the change of affordability of health care and other goods and services) during the economic boom (recession) period. It is worthy addressing that previous studies [see Liu et al. (49) for recent example] could not distinguish health impacts due to positive and negative economic shocks. The Hypothesis 3 (Asymmetric health cycles hypothesis) is the one hypothesis that could justify the asymmetric effect of business cycles on population health. Moreover, our model specification distinguishes the asymmetric effects of business cycles on population health in the long- and short-run from co-integrating equation (Equation 3) and the impulse-response analyses(in terms of the responses of population health to positive or negative economic shocks), respectively.

### Data Sources

The historical real GDP per capita and life expectancy at birth for the ASEAN countries were obtained from the Maddison Project Database, 2018 (administrated by the University of Groningen, the Netherlands) and Gapminder Database (maintained by the Gapminder Foundation in Stockholm), respectively. The sample period starts from 1950 and ends in 2016, resulting in a total of 67 annual observations for each country. In order to calculate the cumulative positive and negative sums of ln(LE) (extracted from Gapminder Database) and ln(GDP) (obtained from Maddison Project Database), the 1950 data served as initial period observations and were dropped from our analyses. Therefore, there are 66 annual observations for each country included in our analyses.

## Results

### Descriptive Statistics

**Figure 1:**
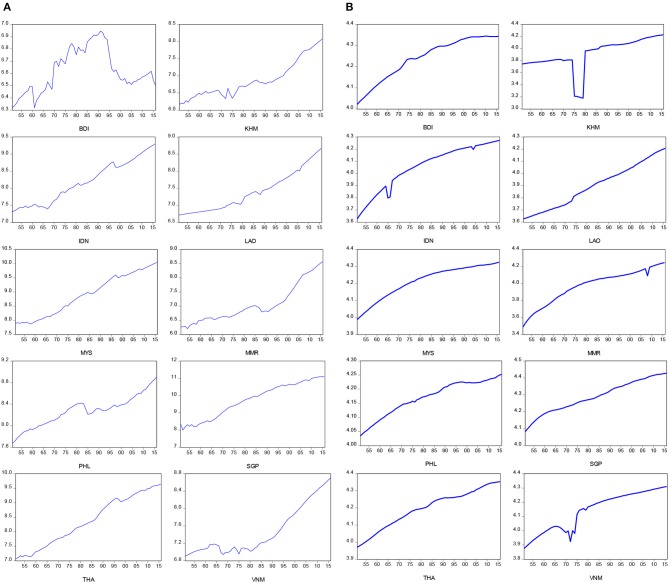
Time plots for real GDP per capita and life expectancy at birth. **(A)** Real GDP per capita in log-scale. **(B)** Life expectancy at birth in log-scale.

**Figure 2:**
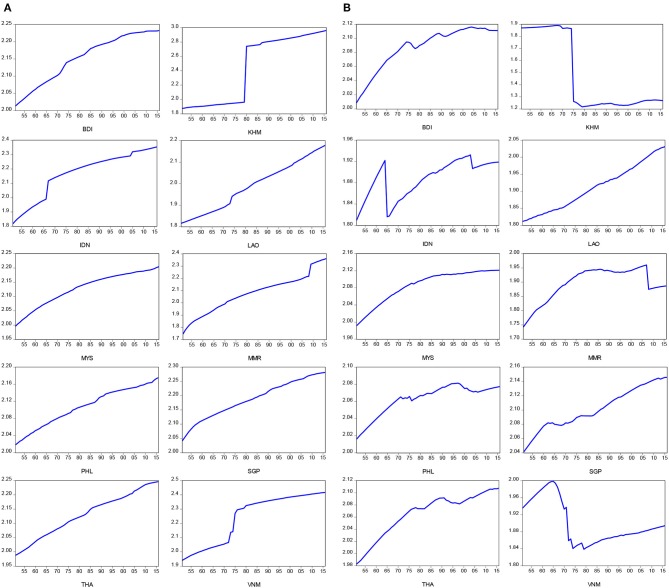
Time plots for positive and negative components for life expectancy at birth. **(A)** Positive component for LE at birth in log-scale. **(B)** Negative component for LE at birth in log-scale.

**Figure 3:**
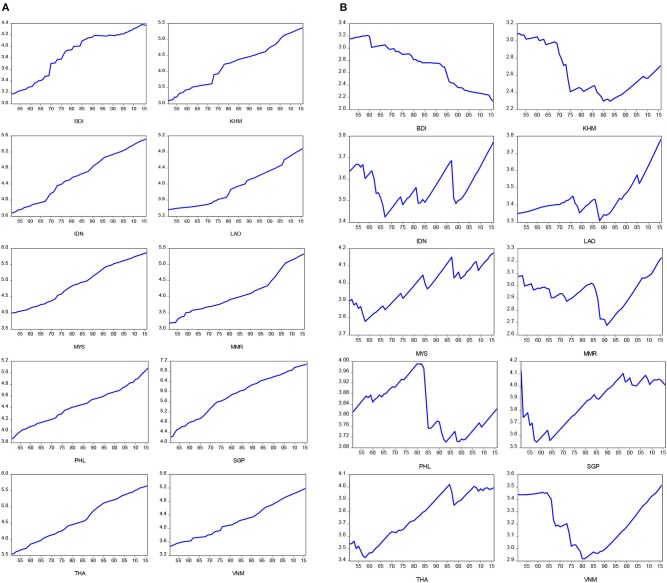
Time plots for positive and negative components for real GDP per capita. **(A)** Positive component for real GDP per capita in log-scale. **(B)** Negative component for real GDP per capita in log-scale.

Table 1 displays the descriptive statistics of life expectancy at birth and real GDP per capita across the ASEAN countries over the period of 1950–2016. In order to better illustrate our data, the time plots of all variables used in this study are presented in Figures 1–3. Figure 1 shows a positively deterministic trend, in general, in life expectancy at birth and real GDP per capita across the entire sample, and based on these results, we decomposed the positive and negative components of the variables. In general GDP per capita showed an upward trend during the same period, but several downturn points reflect bad years such as the periods of two oil crises (1973–1975 and 1980–1982) for Brunei (BDI), Cambodia (KHM), Indonesia (IDN), Laos (LAO), Philippines (PHL), and Vietnam (VNM), the 1990s recession (1990–1992) for Cambodia (KHM), Laos (LAO), and Philippines (PHL), Asian financial crisis (1999–2000) for Brunei (BDI), Indonesia (IDN), and Thailand (THA), and the subprime loan crisis (2007–2009) for Cambodia (KHM), Laos (LAO), Myanmar (MMR), Philippines (PHL), Singapore (SGP), and Thailand (THA). Moreover, the life expectancy at birth showed the same upward trend during the same period, but several downturn points reflect some transitional years in politics such the periods of the Vietnam War (1965–1975) for Cambodia (KHM), Myanmar (MMR), Indonesia (IDN), and Vietnam (VNM), and the 2004 Tsunami tragedy for Indonesia (IDN).

**Table 1:** Descriptive statistics.

		**Life expectancy at birth (LE)**				**Real GDP per capita (GDP)**				
**Country**	**Code**	**Mean**	**Std. Dev**.	**Min**.	**Max**.	**Mean**	**Std. Dev**.	**Min**.	**Max**.	**Obs**.
Brunei	BDI	69.566	6.788	55.500	77.100	759.239	138.786	536.000	1039.000	67
Indonesia	IDN	58.257	10.257	37.200	71.700	4246.970	2632.262	1410.000	10911.000	67
Cambodia	KHM	51.512	11.463	24.000	68.700	1122.433	724.702	472.000	3251.000	67
Laos	LAO	49.970	9.106	37.500	67.300	1932.985	1307.462	812.000	5859.000	67
Myanmar	MMR	53.779	10.242	31.900	70.000	1477.030	1256.108	466.000	5284.000	67
Malaysia	MYS	67.712	6.464	53.600	75.600	9239.612	6250.903	2624.000	23053.000	67
Philippines	PHL	64.710	3.987	56.300	70.300	4029.328	1184.098	2005.000	7410.000	67
Singapore	SGP	72.997	6.963	58.800	83.700	25292.750	20306.640	2951.000	65729.000	67
Thailand	THA	66.627	7.367	52.800	77.800	5897.940	4512.289	1140.000	15454.000	67
Vietnam	VNM	63.151	8.477	47.900	74.500	2074.418	1395.655	971.000	6062.000	67
All		61.828	11.274	24.000	83.700	5607.270	9858.483	466.000	65729.000	670
		**ln(LE)**				**ln(GDP)**				
Brunei	BDI	4.241	0.098	4.022	4.345	6.621	0.176	6.317	6.946	66
Indonesia	IDN	4.055	0.183	3.627	4.272	8.181	0.613	7.309	9.298	66
Cambodia	KHM	3.915	0.260	3.178	4.230	6.877	0.528	6.165	8.087	66
Laos	LAO	3.899	0.180	3.627	4.209	7.396	0.578	6.712	8.676	66
Myanmar	MMR	3.973	0.198	3.487	4.248	7.055	0.662	6.203	8.572	66
Malaysia	MYS	4.214	0.096	3.987	4.325	8.898	0.725	7.872	10.046	66
Philippines	PHL	4.170	0.061	4.034	4.253	8.270	0.282	7.676	8.911	66
Singapore	SGP	4.289	0.094	4.083	4.427	9.739	0.998	7.990	11.093	66
Thailand	THA	4.196	0.111	3.970	4.354	8.371	0.844	7.077	9.646	66
Vietnam	VNM	4.140	0.136	3.875	4.311	7.474	0.554	6.906	8.710	66
All		4.109	0.202	3.178	4.427	7.888	1.126	6.165	11.093	660
		**ln(LE)**^**+**^				**ln(GDP)**^**+**^				
Brunei	BDI	2.153	0.069	2.013	2.233	3.882	0.401	3.167	4.383	66
Indonesia	IDN	2.167	0.158	1.818	2.354	4.606	0.585	3.673	5.524	66
Cambodia	KHM	2.440	0.467	1.874	2.961	4.236	0.683	3.084	5.371	66
Laos	LAO	1.989	0.113	1.816	2.178	3.950	0.486	3.361	4.887	66
Myanmar	MMR	2.078	0.154	1.745	2.362	4.105	0.618	3.189	5.341	66
Malaysia	MYS	2.128	0.058	1.996	2.204	4.915	0.619	3.996	5.870	66
Philippines	PHL	2.107	0.045	2.018	2.175	4.442	0.317	3.864	5.086	66
Singapore	SGP	2.188	0.066	2.041	2.282	5.871	0.843	4.225	7.086	66
Thailand	THA	2.131	0.077	1.988	2.247	4.610	0.658	3.540	5.650	66
Vietnam	VNM	2.241	0.173	1.940	2.417	4.249	0.526	3.468	5.191	66
All		2.162	0.213	1.745	2.961	4.487	0.806	3.084	7.086	660
		**ln(LE)**^**−**^				**ln(GDP)**^**−**^				
Brunei	BDI	2.088	0.030	2.008	2.116	2.739	0.335	2.134	3.210	66
Indonesia	IDN	1.888	0.035	1.809	1.932	3.575	0.083	3.425	3.774	66
Cambodia	KHM	1.475	0.307	1.217	1.890	2.641	0.269	2.296	3.085	66
Laos	LAO	1.911	0.067	1.811	2.031	3.445	0.112	3.309	3.788	66
Myanmar	MMR	1.895	0.058	1.742	1.960	2.950	0.123	2.677	3.232	66
Malaysia	MYS	2.086	0.038	1.991	2.121	3.983	0.111	3.779	4.176	66
Philippines	PHL	2.063	0.017	2.016	2.081	3.828	0.088	3.701	3.991	66
Singapore	SGP	2.102	0.028	2.041	2.146	3.867	0.180	3.545	4.129	66
Thailand	THA	2.066	0.035	1.983	2.108	3.762	0.193	3.426	4.020	66
Vietnam	VNM	1.899	0.050	1.839	1.998	3.225	0.194	2.917	3.519	66
All		1.947	0.208	1.217	2.146	3.402	0.500	2.134	4.176	660

*The whole sample period starts from 1950 and ends in 2016, resulting in a total of 67 annual observations for each country. In order to calculate cumulative positive and negative sums of ln(LE) and ln(GDP), the 1950 data are treated as initial period observations and dropped from our analyses. Therefore, there are 66 annual observations for each country included in our analyses*.

Figures 2A,B present time plots of the positive and negative components of life expectancy at birth, respectively. We found that the significant breaks of positive and negative components of life expectancy at birth appear during the periods of the Vietnam War (1965–1975) for Cambodia (KHM), Myanmar (MMR), Indonesia (IDN), and Vietnam (VNM), and the subprime loan crisis (2007–2009) for Laos (LAO). In addition, Figures 3A,B present time plots of the positive and negative components of real GDP per capita, respectively. The significant breaks of positive component of real GDP per capita were found during the periods of two oil crises (1973–1975, and 1980–1982) for Brunei (BDI), Cambodia (KHM), Indonesia (IDN), and Laos (LAO), while the significant breaks of negative component of real GDP per capita were identified found during the periods of the Vietnam War (1965–1975) for Cambodia (KHM), Myanmar (MMR), Indonesia (IDN), and Vietnam (VNM), the 1990s recession (1990–1992) for Laos (LAO), Myanmar (MMR), Philippines (PHL), and Asian financial crisis (1999–2000) for Brunei (BDI), Indonesia (IDN), Malaysia (MYS), Singapore (SGP), and Thailand (THA). In sum, the upward trends of the negative components of life expectancy at birth were found in almost all ASEAN countries, except for Cambodia (KHM) and Vietnam (VNM). The negative components of real GDP per capita showed upward trends in Laos (LAO), Malaysia (MYS), and Thailand (THA) and downward trends in Brunei (BDI) and Cambodia (KHM). The negative components of real GDP per capita exhibited a U-shape in Indonesia (IDN), Myanmar (MMR), Singapore (SGP), and Vietnam (VNM) and an inverted U-shape in Philippines (PHL).

### Panel Unit Root Tests

Table 2 presents the results from the panel unit root test for life expectancy at birth, real GDP per capita, and their positive and negative components in level terms. As indicated in Table 2, all the Breitung *t* statistics (generating *p* <1%) fail to reject the null hypothesis of panel unit roots of all variables in level terms but they provided significant evidence of stationarity of the first difference. These findings indicate that all variables used for our analyses belong to the I(1) series, so panel cointegration tests were performed in order to justify the cointegrated relationship among the variables of interest.

**Table 2:** Panel unit root tests.

**Variables**	**Level**		**1st Diff**	
	**Statistic**	***P*-value**	**Statistic**	***P*-value**
ln(LE)	5.165	1.00	**−6.170**	0.00^**^
ln(LE)^+^	4.032	1.00	**−7.019**	0.00^**^
ln(LE)^−^	4.939	1.00	**−8.994**	0.00^**^
ln(GDP)	2.240	0.99	**−10.346**	0.00^**^
ln(GDP)^+^	1.934	0.97	**−8.173**	0.00^**^
ln(GDP)^−^	3.003	1.00	**−8.589**	0.00^**^

Breitung t-statistics were reported, and other panel unit root tests such as such as the LLC test, IPS test, and ADF-Fisher and PP-Fisher chi-square tests are available upon request to authors. Bold font represents the rejection of 1% significance levels.

** *signifies 1% significance level*.

### Panel Cointegration Tests

Table 3 shows the results of two panel cointegration tests which justify a possible cointegrated relationship among the variables. Surprisingly, the cointegration relationship between life expectancy at birth and real GDP per capita cannot be verified by the Kao panel cointegration test, as we failed to reject the null hypothesis of no cointegrated relationship between life expectancy at birth and real GDP per capita (see Panel A of Table 3). We resorted to Hatemi (26)'s methodology, considering the possibility of a hidden cointegration relationship, by specifying either GDP per capita as a function of the positive and negative components of life expectancy at birth or life expectancy at birth as a function of the positive and negative components of real GDP per capita. Once again, the Kao panel cointegration test failed to reject the null hypothesis of no cointegrated relationship between GDP per capita as a function of the positive and negative components of life expectancy at birth. Nevertheless, the cointegrated relationship between life expectancy at birth and the positive and negative components of real GDP per capita is proved by the rejection of the null hypothesis of no cointegration based on the Kao panel cointegration test.

**Table 3:** Panel cointegration tests.

**Panel A: Kao panel cointegration test**	**Hypothesis**	**ADF**		***P*****-value**	
ln(LE), ln(GDP)	H_0_: No Cointegration	−1.641		0.05	
ln(GDP), ln(LE)^+^, ln(LE)^−^	H_0_: No Cointegration	−0.348		0.36	
ln(LE), ln(GDP)^+^, ln(GDP)^−^	H_0_: No Cointegration	−2.538		0.00^**^	
**Panel B: Johansen Fisher panel cointegration tests**	**No. of cointegration equations**	**Trace test Fisher-stat**	***P*****-value**	**Max-Eigen value test Fisher-stat**	***P*****-value**
ln(LE), ln(GDP)^+^, ln(GDP)^−^	H_0_: No Cointegration	46.946	0.00^**^	36.376	0.00^**^
	H_0_: At most 1	10.571	0.43	10.527	0.35

** *signifies 1% significance level*.

We then continued with the selection of the number of cointegrated relationships between life expectancy at birth and the positive and negative components of real GDP per capita based on the Johansen Fisher panel cointegration tests. As indicated in Panel B of Table 3, the Fisher statistics generated by both trace and maximum eigenvalue tests reject the null hypothesis of no cointegrated relationship between life expectancy at birth and the positive and negative components of real GDP per capita but they failed to reject the null hypothesis for at least one relationship among these three variables of interest. The evidence obtained from both Kao and Johansen Fisher panel cointegration tests suggests that there is one possible long-run relationship among life expectancy at birth and the positive and negative components of real GDP per capita.

### Panel Vector Error Correction Model

The panel vector error correction model was estimated together with the Johansen Fisher panel cointegration test, and the estimated results are shown in Table 4. As indicated in the table, the estimated coefficient is significant for the positive component of real GDP per capita but it is not significant for the negative component. Together, the hidden cointegrated relationship between life expectancy at birth and the positive and negative components of real GDP per capita identified by the panel cointegration tests indicate a long-run asymmetric effect of business cycles on population health.

**Table 4:** Panel vector error correction model.

	**Δln(LE)_**it**_**	**Δ${\text{ln(GDP)}}_{\text{it}}^{+}$**	**Δln(GDP)${\text{ln(GDP)}}_{\text{it}}^{-}$**
**Short-run**			
Δln(LE)_it−1_	−0.001	−0.016	−0.041
	(−0.032)	(−0.684)	(−1.422)
Δln(LE)_it−2_	0.028	−0.016	0.013
	(0.720)	(−0.691)	(0.446)
Δln(GDP)${\text{ln(GDP)}}_{\text{it}-1}^{+}$	−0.064	0.234	−0.025
	(−0.918)	(5.447)	(−0.483)
Δ${\text{ln(GDP)}}_{\text{it}-2}^{+}$	**−0.283**	**0.091**	−0.041
	**(**–4.186)^**^	(2.181)^*^	(−0.824)
Δ${\text{ln(GDP)}}_{\text{it}-1}^{-}$	**0.200**	**−0.072**	**0.303**
	(3.450)^**^	**(**–2.024)^*^	(7.058)^**^
Δ${\text{ln(GDP)}}_{\text{it}-2}^{-}$	0.044	−0.045	0.040
	(0.833)	(−1.369)	(1.013)
Constant	**0.018**	**0.017**	−0.003
	(4.696)^**^	(7.011)^**^	(−1.004)
Trend(×10^−3^)	−0.005	0.009	**0.002**
	(−0.649)	(1.677)	(2.650)^**^
**Long-run**	**ln(LE)it**	**ln(GDP)**_**+it**_	**ln(GDP)**_**−it**_
Cointegration vector (β)	1.000	–**0.183**	0.089
		**(**–5.374)^**^	(1.574)
Adjustment coefficient (α)	–**0.066**	−0.013	−0.007
	**(**–5.786)^**^	(−1.91]	(−0.858)
Long-run relationship: ln(LE)_it_ = 3.538 + 0.164 × Trend(×10^−3^) + 0.183 × ${\text{ln(GDP)}}_{\text{it}}^{+}$ − 0.089 × ${\text{ln(GDP)}}_{\text{it}}^{-}$+ e_it_			
**Long-run asymmetric test**	**Statistic**		
Ho: symmetric effect	**χ^2^ = 9.056**		
H_1_: asymmetric effect	***P***-value = 0.00^**^		
Short-run asymmetric test	Statistic		
Ho: symmetric effect	**χ^2^ = 16.541**		
H_1_: asymmetric effect	***P***-value = 0.00^**^		

t-statistics were displayed in the brackets, and

** and

* signify 1 and 5% significance levels, respectively.

χ2 for the Short-Run Asymmetric Test was obtained by restricting the sum of coefficients of positive components of ln(GDP) to be the same as the sum of coefficients of negative components of ln(GDP) based on the single equation VECM model.

*Bold values represent variables that are significant at 1% or 5% level*.

We formally tested the null hypothesis of the long-run symmetric effect of business cycles on population health (namely, Ho:β^+^ = β^−^) using the Wald test. The Chi-square statistic generated by the test is 9.056 with a *p*-value at less than a 1% significance level, suggesting a result in favor of the long-run asymmetric effect of business cycles on population health. The value of the estimated coefficient for the positive component of real GDP per capita is 0.183, meaning that a 1% increase in the positive component of real GDP per capita (due to a positive economic shock) will expand longevity by 0.183%.

Since this study focuses on the effect of business cycles on population health, our interpretation of results relies on the results displayed in the second column of Table 4. The estimated adjustment coefficient is significantly negative, and this result suggests that the short-run deviation from the long-run path of life expectancy at birth will eventually adjust toward the long-run equilibrium for population health. In addition, we found that the signs of all estimated coefficients for the difference of positive components of real GDP per capita are negative but they are positive for the difference of negative components. These results also suggest a short-run asymmetric effect of business cycles on population health. Once again, we formally tested the null hypothesis of the short-run symmetric effect (namely, Ho: $\pi_{\Delta \ln\;\left(GDP\right),t-1}^{+}+\pi_{\Delta \ln\;\left(GDP\right),t-2}^{+}=\pi_{\Delta \ln\;\left(GDP\right),t-1}^{-}+\pi_{\Delta \ln\;\left(GDP\right),t-2}^{-}$) using the Wald tests. The Chi-square statistic generated by the Wald test is 16.541 with a *p*-value at less than a 1% significance level, suggesting a result in favor of the short-run asymmetric relationship between business cycles (measured by the log-difference of real GDP per capita) and health progress (measured by log-difference of life expectancy at birth).

### Pairwise Granger Causality

In order to interpret the results of the impulse-response analyses, the pairwise Granger causality tests were used to identify the lead-lag relationship between life expectancy at birth and the positive and negative components of real GDP per capita. As shown in Table 5, one-way causal linkage was identified by the pairwise Granger causality tests for three pairs of variables: causal linkage running from the positive component of real GDP per capita to life expectancy at birth, causal linkage running from the negative component of real GDP per capita to life expectancy at birth, and causal linkage running from the positive component to the negative component of real GDP per capita over the study period. These results confirm that our co-integrating specifying life expectancy at birth as the dependent variable and real GDP per capita as the independent variable is correct. We restricted our analyses on the causal relationship between life expectancy at birth and the positive (negative) components of real GDP per capita since these two relationships are most closely related to the purpose of our study.

**Table 5:** Pairwise Granger causality tests.

			***F*-Statistic**	***P*-value**
H_0_: Δln(GDP)^+^	≠ >	Δln(LE)	6.026	0.00^**^
H_0_: Δln(LE)	≠ >	Δln(GDP)^+^	1.203	0.30
H_0_: Δln(GDP)^+^	≠ >	Δln(LE)	5.316	0.00^**^
H_0_: Δln(LE)	≠ >	Δln(GDP)^+^	1.278	0.28
H_0_: Δln(GDP)^−^	≠ >	Δln(GDP)^+^	4.472	0.01^*^
H_0_: Δln(GDP)^+^	≠ >	Δln(GDP)^−^	0.295	0.74

** and

* *signify 1 and 5% significance levels, respectively*.

### Impulse-Response Analyses

**Figure 4:**
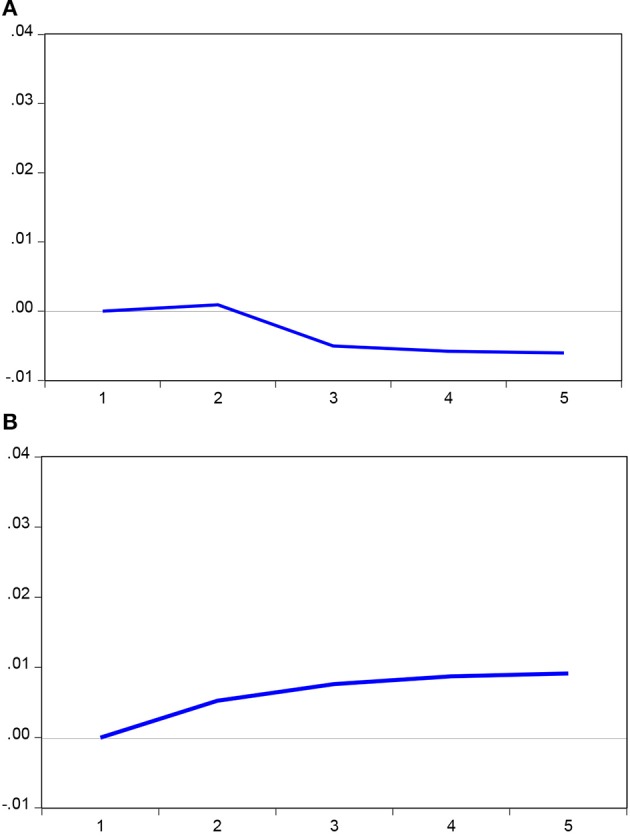
Response to Cholesky one SD innovation. **(A)** Response of ln(LE) to ln(GDP)^+^. **(B)** Response of ln(LE) to ln(GDP)^−^.

The short-run effect of the asymmetric effect of business cycles on population health is further illustrated in the results of the impulse-response analyses. Both positive and negative components of real GDP per capita appear to lead life expectancy at birth based on the results of the pairwise Granger causality tests shown in Table 5, so we present the propagation mechanism of an economic shock over a period of time through the impulse-response analyses in Figure 4. Figure 4A illustrates the response of life expectancy at birth to one standard increase in the positive components of real GDP per capita over a 5-year horizon. An increase in the positive components of real GDP per capita (due to a positive economic shock) leads to a positive change in life expectancy at birth only in the first year, but it results in a negative change in life expectancy at birth for the remaining 4 years. Figure 4B demonstrates the response of life expectancy at birth to one standard decrease in the negative components of real GDP per capita. A decrease in the negative components of real GDP per capita (due to a negative economic shock) leads to an increase change in life expectancy at birth over our observed 5-year horizon. The findings suggest that both economic boom and economic recession affect population health asymmetrically in the short run. In general, the positive effect of economic recession on population health dominates over the 5 years horizon.

## Discussion

Previous empirical findings have found that the relationship between business cycles and population health could be positive (27–29, 57), negative (5–8, 14), or unrelated (37). However, the ambiguous relationship may result from the fact that business cycles may have asymmetric effects on population health. In this study, we decompose the positive and negative components of real GDP per capita and apply panel cointegration tests, Granger causality tests, and impulse-response analyses based on the PVECM to examine the existence of the asymmetric effects of business cycles on population health. Based on the results of the PVECM, the null hypotheses of the long- and short-run symmetric effects of business cycles on population health were rejected in our specification of PVECM, providing the evidence of asymmetric effects of business cycles on population health. It follows that Hypothesis 1 (Asymmetric health cycles hypothesis) is supported by our data across ASEAN countries from the period of 1951–2016. In addition, we notice that the estimated coefficient for the positive component of real GDP per capita (β^+^) in Equation (3) is positive but the estimated coefficient for the negative component of real GDP per capita (β^−^) is negative. The former is statistically significant at 1% significance level but the latter is statistically insignificant. Namely, an economic boom (i.e., increase in the positive component of real GDP per capita) has a positive impact on life expectancy at birth, but an economic recession (i.e., decrease in the negative component of real GDP per capita) insignificantly affects life expectancy at birth so that the Hypothesis 2 (Pro-cyclicality of population health to business cycles hypothesis) is weakly consistent with our data in the long-run.

Nevertheless, we found that over a 5-year horizon, the impulse-response analyses indicate that a positive economic shock leads to a positive change in life expectancy in the first year, but decreases the life expectancy at birth for years 2–5. In addition, a negative economic shock leads to a positive change in life expectancy for the entire 5-year horizon. These results recommend that the Hypothesis 3 (Counter-cyclicality of population health to business cycles hypothesis) could justified in the short-run across ASEAN countries from the period of 1951–2016. Therefore, evidence generated from our asymmetric empirical model harmonizes unsettled arguments raised by the pro-cyclicality of population health and counter-cyclicality of population health to business cycles hypotheses. Our results identified the asymmetric effects of business cycles on population health both in the long run and in the short run. We propose that failure to consider the asymmetric effects in empirical model specifications may lead to a biased relationship between business cycles and population health.

Several policy implications based on our findings have their merits to be addressed:

First, despite the political and economic institutional settings of ASEAN countries differ from those of the European countries (14), OECD member countries (10), and the Asia-Pacific region (11), the establishment of the pro-cyclicality of population health to business cycles hypothesis in the long-run across ASEAN countries is consistent with the economic growth theory grounded by the experiences from advanced economies (41, 44). Economic growth allows governments to increase public health and education expenditures and provide more public health services that would accumulate health capital, and in turn benefit population health.
Second, economic development may lead to changes in health behaviors in various ways. During an economic boom, people with increasing incomes can consume more nutritious foods, which is particularly beneficial for better health outcomes and longer life expectancy in emerging economies such as the ASEAN countries. Furthermore, economic development allows governments to increase public health care expenditures and provide more public health services. However, an economic improvement does not necessarily give rise to good health. The so-called “diseases of affluence,” the illnesses and diseases linked with economic prosperity, become prevalent as societies advance along the stages of economic development. Previous studies have found that people may increase alcohol and smoking consumption during economic expansions (7, 58–60). Adverse lifestyle habits, job-related stress, extended working hours, and less time spent on exercise may, in turn, result in an increase in non-communicable diseases such as obesity, diabetes, and other chronic ailments. The negative effects outweigh the positive effects which lead to a decrease in life expectancy during the latter years of an economic boom. The establishment of the counter-cyclicality of population health to business cycles hypothesis in the short-run across ASEAN countries reflect the phenomenon of the diseases of affluence due to accelerating economic growth in the short-run.
Third, the interventions for the health promotion should be taken differently during the economic boom and recession periods based on our findings on the asymmetric effects of business cycles on population health. During periods of economic expansion, governments should put more emphasis on increasing public awareness and understanding of health issues that may accompany rapid economic development in emerging countries and methods for improving health outcomes. Although the individual is more likely to increase health promotion activities due to the low opportunity cost of health promotion during the periods of economic recession. Nevertheless, Liang and Tussing (61) indicate show that cutting back on government health expenditure in bad times could result in undersupply of public services that are most crucial when household incomes drop. Arbitrary cuts to health services may further worsen the health system if they erode access to healthcare and the quality of care provided, increasing health and other costs. Therefore, during periods of economic recession, governments should implement effective programs and services to mitigate the harmful effects on population health, especially for individuals facing lower household income and certain forms of psychosocial stress as a result of job loss.

This research has several contributions on the literature of business cycles on population health. First, this paper extends the existing empirical evidence (mainly based on advanced economies) by investigating the effect of business cycles on population across ASEAN countries for the first time. Second, the hidden cointegration methodology introduced by Hatemi (26) was used to model the asymmetric effects of business cycles on population health in both long- and short-run. The asymmetric effects identified by our empirical model fill the gap of previous studies using the individual time series of advanced economies (9, 21, 22, 28, 29, 49) and reconcile results from prior studies that document a pro-cyclical pattern between business cycles and population health (27–29, 57) with other studies suggesting the changing relationship between business cycles and population health (12, 21, 22, 36, 49).

This study, nevertheless, suffers from several limitations: First, our cointegrating analyses restricted in the bivariate type of analyses because the time series data regarding socio-economic variables other than real GDP per capita and life expectancy at birth across the ASEAN countries is too short to perform a reliable time series analyses. Second, due to the limited time series data for the variables used for our analyses, we intend to draw a reliable conclusion of the asymmetric effect of business cycles on population health from a group of high-growth emerging economy, namely ASEAN. We demand longer time series data to investigate the asymmetric effect of business cycles on population health in the future.

## Conclusion

The relationship between population health and business cycles has been inconclusive in the previous studies. Some studies using data from more advanced economies indicated that population health is counter-cyclical with business cycles, while others suggested otherwise. Few studies have examined the effect of business cycles on population health in emerging economies. In order to fill this gap in the literature, we have followed the hidden cointegration methodology proposed by Hatemi (26) to investigate the effect of business cycles on population over the period of 1951–2016 across the ASEAN countries (i.e., Brunei, Cambodia, Indonesia, Laos, Malaysia, Myanmar, Philippines, Singapore, Thailand, and Vietnam) through the PVECM.

Our results suggest that there is a hidden cointegrated relationship between life expectancy at birth and the positive and negative components of real GDP per capita. The asymmetric effects of business cycles on population health were identified in both short and long-run through the PVECM. Specifically, we found that the population health is pro-cyclical to business cycles in the long-run but it is counter-cyclical to business cycles in the short-run. Therefore, not only economic boom may have a significantly positive effect on population health in the long run, but economic recession also has positive effects on population health in the short run. These findings are consistent with the economic growth theory of human capital (41) and health behavior pathway model (22). Policymakers should focus on the harmful effects of business cycles on population health, and government interventions should be more forceful in times of economic expansion than in economic recession periods.

## Data Availability Statement

The datasets generated for this study are available on request to the corresponding author.

## Author Contributions

All authors have planned and contributed in writing the manuscript. All authors have critically reviewed and revised the manuscript and approved the final product.

### Conflict of Interest

The authors declare that the research was conducted in the absence of any commercial or financial relationships that could be construed as a potential conflict of interest.
