# Supplementary material for: Asymmetric Effect of Business Cycles on Population Health: Evidence From the ASEAN Countries
Source: Front Public Health. 2020 Feb 21;8:32. doi: 10.3389/fpubh.2020.00032 (PMC7047288; doi:10.3389/fpubh.2020.00032)
Supplement: Supplementary file 1 [file Data_Sheet_1.docx]

**Appendix**

In order to model the cointegrated relationship between population health (measured by life expectancy at birth) and economic development (measured by real GDP per capita) with a deterministic trend, we define these two variables based on Hatemi-J (2014), Hatemi-J and El-Khatib (2016), and Hatemi-J (2014) as follows:

[A1] $\left\{\begin{matrix} \ln(LE)_{i,t}=a_{i}+b_{i}t+\ln(LE)_{i,t-1}+e_{i1,t}=\ln(LE)_{i,0}+\sum_{j=1}^{t} e_{i1,j} \\ \ln(GDP)_{i,t}=c_{i}+d_{i}t+\ln(GDP)_{i,t-1}+e_{i2,t}=\ln(GDP)_{i,0}+\sum_{j=1}^{t} e_{i2,j} \end{matrix}\right.$

where $LE_{i,t}$ and $GDP_{i,t}$ represent life expectancy at birth and GDP per capita, respectively. Subscript *i*=1,2,3,…,*N* denotes the individual country, and *N* represents the total number of countries included in our study. $a_{i}$ and $c_{i}$ are the drifts, and *t*=1,2,3,…,*T* is the deterministic trend component. $b_{i}$ and $d_{i}$ are coefficients corresponding to the deterministic trend components for $\ln(LE)_{i,t}$ and $\ln(GDP)_{i,t}$. $e_{ih,t}$ (for *h*=1,2) is white noise residual, and $\sum_{j=1}^{t} e_{ih,j}$(for *h*=1,2) is the cumulative sum of the residuals. In order to model the asymmetric cointegrated relationship between $\ln(LE)_{i,t}$ and $\ln(GDP)_{i,t}$, the positive and negative shocks for these two panel variables are defined as follows:

[A2] $\left\{\begin{matrix} e_{i1,t}^{+}:=Max(e_{i1,t},0)\text{ ;} & e_{i1,t}^{-}:=Min(e_{i1,t},0) \\ e_{i2,t}^{+}:=Max(e_{i2,t},0)\text{ ;} & e_{i2,t}^{-}:=Min(e_{i2,t},0) \end{matrix}\right.$

It follows that the positive and negative components could be defined as follows:

[A3] $\left\{\begin{matrix} \ln(LE)_{i1,t}^{+}=\frac{a_{i}t+(t(t+1)/2)b_{i}+\ln(LE)_{i,0}}{2}+\sum_{j=1}^{t} e_{i1,j}^{+} \\ \ln(LE)_{i1,t}^{-}=\frac{a_{i}t+(t(t+1)/2)b_{i}+\ln(LE)_{i,0}}{2}+\sum_{j=1}^{t} e_{i1,j}^{-} \\ \ln(GDP)_{i2,t}^{+}=\frac{a_{i}t+(t(t+1)/2)b_{i}+\ln(GDP)_{i,0}}{2}+\sum_{j=1}^{t} e_{i2,j}^{+} \\ \ln(GDP)_{i2,t}^{-}=\frac{a_{i}t+(t(t+1)/2)b_{i}+\ln(GDP)_{i,0}}{2}+\sum_{j=1}^{t} e_{i2,j}^{-} \end{matrix}\right.$

where $\ln(LE)_{i1,t}$=$\ln(LE)_{i1,t}^{+}$+$\ln(LE)_{i1,t}^{-}$ and $\ln(GDP)_{i1,t}$=$\ln(GDP)_{i1,t}^{+}$+$\ln(GDP)_{i1,t}^{-}$. $\sum_{j=1}^{t} e_{ih,j}^{+}$ and $\sum_{j=1}^{t} e_{ih,j}^{-}$ (for *h*=1,2) are positive and negative cumulative sum of shocks, respectively.
